# Effects of caffeine on neuromuscular fatigue and performance during high-intensity cycling exercise in moderate hypoxia

**DOI:** 10.1007/s00421-016-3496-6

**Published:** 2016-11-18

**Authors:** Bruno P. C. Smirmaul, Antonio Carlos de Moraes, Luca Angius, Samuele M. Marcora

**Affiliations:** 1 Department of Physical Education, São Paulo State University (UNESP), Rio Claro, SP Brazil; 2 Faculty of Physical Education, University of Campinas (UNICAMP), São Paulo, SP Brazil; 3 Endurance Research Group, School of Sport and Exercise Sciences, University of Kent at Medway, Chatham Maritime, Kent ME4 4AG UK

**Keywords:** Altitude, Perception of effort, Central fatigue, Peripheral fatigue, Exercise performance

## Abstract

**Purpose:**

To investigate the effects of caffeine on performance, neuromuscular fatigue and perception of effort during high-intensity cycling exercise in moderate hypoxia.

**Methods:**

Seven adult male participants firstly underwent an incremental exercise test on a cycle ergometer in conditions of acute normobaric hypoxia (fraction inspired oxygen = 0.15) to establish peak power output (PPO). In the following two visits, they performed a time to exhaustion test (78 ± 3% PPO) in the same hypoxic conditions after caffeine ingestion (4 mg kg^−1^) and one after placebo ingestion in a double-blind, randomized, counterbalanced cross-over design.

**Results:**

Caffeine significantly improved time to exhaustion by 12%. A significant decrease in subjective fatigue was found after caffeine consumption. Perception of effort and surface electromyographic signal amplitude of the vastus lateralis were lower and heart rate was higher in the caffeine condition when compared to placebo. However, caffeine did not reduce the peripheral and central fatigue induced by high-intensity cycling exercise in moderate hypoxia.

**Conclusion:**

The caffeine-induced improvement in time to exhaustion during high-intensity cycling exercise in moderate hypoxia seems to be mediated by a reduction in perception of effort, which occurs despite no reduction in neuromuscular fatigue.

## Introduction

It is well established that caffeine reduces perception of effort and improves performance during endurance exercise at sea level (reviewed in Graham 2001; Doherty and Smith 2004, 2005; Goldstein et al. 2010), and a large proportion of endurance athletes consume it before or during competitions (Del Coso et al. 2011). Little is known, however, about the ergogenic effect of caffeine in conditions of hypoxia. This is surprising if we consider that some endurance competitions are staged at high altitude (e.g., Tour de France) and hypoxia has a negative effect on endurance performance (reviewed in Amann and Calbet 2008). Physical endurance is also required by mountaineers and soldiers operating at high altitude. Therefore, a better understanding of the physiological, perceptual, and performance effects of caffeine during endurance exercise in hypoxic conditions is warranted.

To the best of our knowledge, only three investigations have been published on the effects of caffeine on endurance performance in conditions of hypoxia (Berglund and Hemmingsson 1982; Fulco et al. 1994; Stadheim et al. 2015). In the first of these three placebo-controlled studies, Berglund and Hemmingsson (1982) found that caffeine (6 mg kg^−1^) significantly improves performance in 14 well-trained crosscountry skiers competing in a 21-km time trial at 2900 m above sea level. Subsequently, Fulco et al. (1994) confirmed the positive effect of caffeine (4 mg kg^−1^) on endurance performance in conditions of acute hypoxia (4300 m above sea level) by measuring a significant improvement in time to exhaustion in eight young adults cycling at 80% of their altitude-specific maximal oxygen consumption (*V*O_2_max). More recently, Stadheim et al. (2015) tested 13 well-trained cross-country skiers at a simulated altitude of 2000 m above sea level, and found that caffeine (4.5 mg kg^−1^) significantly improves time to exhaustion while double poling at 90% of altitude-specific *V*O_2_max. However, no significant overall improvement in performance was found during an 8-km cross-country double poling time trial.

The improvements in endurance performance observed in these studies were mainly explained in terms of the caffeine-induced reductions in perception of effort and pain (Berglund and Hemmingsson 1982; Fulco et al. 1994; Stadheim et al. 2015). However, we hypothesize that the ergogenic effect of caffeine in hypoxic conditions may also be associated with a reduction in the neuromuscular fatigue induced by endurance exercise. Indeed, hypoxia exacerbates both the central and peripheral fatigue induced by endurance exercise (Amann and Calbet 2008), while caffeine improves neuromuscular function (Kalmar and Cafarelli 2004; Tarnopolsky 2008; Tallis et al. 2015). Several human experiments suggest that caffeine may offset central fatigue by increasing central nervous system (CNS) excitability and maximal voluntary activation (reviewed in Kalmar and Cafarelli, 2004), although more research is necessary to test this hypothesis (Gandevia and Taylor 2006). With regard to peripheral fatigue, it is well established that supraphysiological concentrations of caffeine can reduce fatigue in isolated skeletal muscles, and more recent in vitro studies suggest that physiological concentrations achievable by oral ingestion of caffeine may also directly potentiate skeletal muscle force, work and power (Tallis et al. 2015). For example, caffeine treatment of 70 and 50 μM resulted in significant improvements in power output for both maximally activated mouse extensor digitorum longus (fast) and soleus (slow) muscles (Tallis et al. 2012). In vivo, electrical stimulation studies demonstrate that caffeine can directly improve the contractile properties of fatigued muscles, most likely by potentiating calcium release from the sarcoplasmic reticulum (Lopes et al. 1983; Tarnopolsky and Cupido 2000). Furthermore, in spinal cord-injured patients, caffeine improves time to exhaustion during electrical cycling even though there is no active involvement of the brain, no afferent feedback from leg muscle receptors, and the sympathetic nervous system response is not present (Mohr et al. 1998). This finding suggests that the ergogenic effect of caffeine may occur, at least in part, by its direct action on the active muscles.

A caffeine-induced reduction in neuromuscular fatigue may, in turn, explain at least some of the reduced perception of effort and improved endurance performance found after caffeine ingestion in previous hypoxic studies (Berglund and Hemmingsson 1982; Fulco et al. 1994; Stadheim et al. 2015). In fact, there is mounting evidence that perception of effort is related to central motor command, i.e., the activity of premotor and motor areas of the cortex leading to voluntary muscle contractions (Marcora 2009; Smirmaul 2012; DeMorree et al. 2012, 2014; Zénon et al. 2015). When the neuromuscular system is fatigued, central motor command has to be increased to continue exercising at the same absolute force/power output, leading to an increase in perception of effort and premature exhaustion (Marcora et al. 2008; DeMorree et al. 2012). Importantly, the positive effects of caffeine on the neuromuscular system may partially counteract these negative effects of neuromuscular fatigue by reducing the activity of premotor and motor areas of the cortex required for a given level of submaximal force production, resulting in a lower perception of effort (DeMorree et al. 2014).

The main purpose of this study was to investigate the effects of caffeine on neuromuscular fatigue, perception of effort and performance during high-intensity cycling exercise in conditions of moderate hypoxia equivalent to ≈2500 m altitude. We hypothesize that caffeine ingestion reduces central and peripheral fatigue induced by high-intensity cycling exercise in moderate hypoxia, lowers perception of effort, and increases time to exhaustion.

## Materials and methods

### Participants and ethical approval

A statistical power analysis was performed (GPower 3.1) for sample size estimation based on the time to exhaustion data from a previous hypoxia study (Fulco et al. 1994). As this study did not present correlation values between conditions, a conservative value of 0.5 was used for the calculation. The projected sample size was *N* = 3 with an effect size of 3.68, two-tailed alpha = 0.05 and power = 0.80. Using a more conservative approach, with an effect size of 1.5, the projected sample size was *N* = 6. Thus, seven healthy recreationally active males (mean ± SD, age 29 ± 6 years, height 179 ± 8 cm, weight 75 ± 8 kg, moderate caffeine consumption of 143 ± 77 mg day^−1^, ranging from 49 to 233 mg day^−1^) were recruited for this study. Informed consent, which was approved by the Ethics Committee of the School of Sport and Exercise Sciences according to the standards set by the Declaration of Helsinki, was obtained from all individual participants included in the study. All participants were lifelong sea level residents who had not been exposed to altitudes greater than 1.500 m for at least 6 months before the study.

### Study design

For this study, we employed a double-blind, randomized and counterbalanced cross-over design. Participants visited the laboratory on three different occasions. During the first preliminary visit, participants performed an incremental exercise test in hypoxia and were familiarized with all procedures. During the second and third visit (experimental visits), participants performed neuromuscular tests, and exercised in hypoxia at a constant power output for 6 min (isotime) and then to exhaustion in either a caffeine or placebo condition. Hypoxia during exercise was achieved by filling douglas bags with a gas mixture (HYP-100, Hypoxico Inc., NY, USA) with fraction of inspired oxygen of 15% (FIO_2_ = 0.15). Participants then breathed hypoxic air from the douglas bags using a non-rebreather mask. A minimum of 48 h between visits was given and participants were instructed to refrain from the consumption of any substance containing caffeine/alcohol (24 h prior), to consume a light meal ≈2 h before each visit, as well as to avoid any physical exercise/effort 24 h before each visit. Participants were also asked to inform if they had any acute illness, injury or muscle soreness.

### Procedures

On the first visit, participants completed the Physical Activity Readiness Questionnaire (PAR-Q) (Thomas et al. 1992), a Caffeine Consumption Questionnaire (Landrum 1992) and a *V*O_2max_ Estimation Questionnaire (Bradshaw et al. 2005), and underwent anthropometric measurements. Immediately after a wash-in period of 5-min breathing hypoxic air, an incremental exercise test (3 min at 70 W followed by 1 min of rest and 25 + 25 W increments every minute) was performed in hypoxia until exhaustion [operationally defined as a pedal frequency of less than 70 revolutions^.^min^−1^ (RPM) for more than 5 s despite strong verbal encouragement] on an electromagnetically braked cycle ergometer (Lode BV, Medical Technology, Groningen, The Netherlands) to measure peak power output (PPO), which was calculated according to a published equation (Kuipers et al. 1985). Participants were instructed to maintain a pedal frequency of 70–75 rpm throughout the test. Before the incremental exercise test, the position on the cycle ergometer was adjusted for each participant, and settings were recorded so that they could be reproduced at each subsequent visit. Participants were also given standard instructions for overall perception of effort using the 15-point rating of perceived exertion (RPE) scale (Borg 1982) to be used during the the incremental exercise test and subsequent visits. After the incremental exercise test, participants were familiarized with the neuromuscular assessment procedures (see “Neuromuscular assessment” section) and cycled for ≈6 min at ≈80% of PPO to further adjust the power in which they would exercise during the 6-min isotime period in the next two visits (see below).

**Fig. 1:**
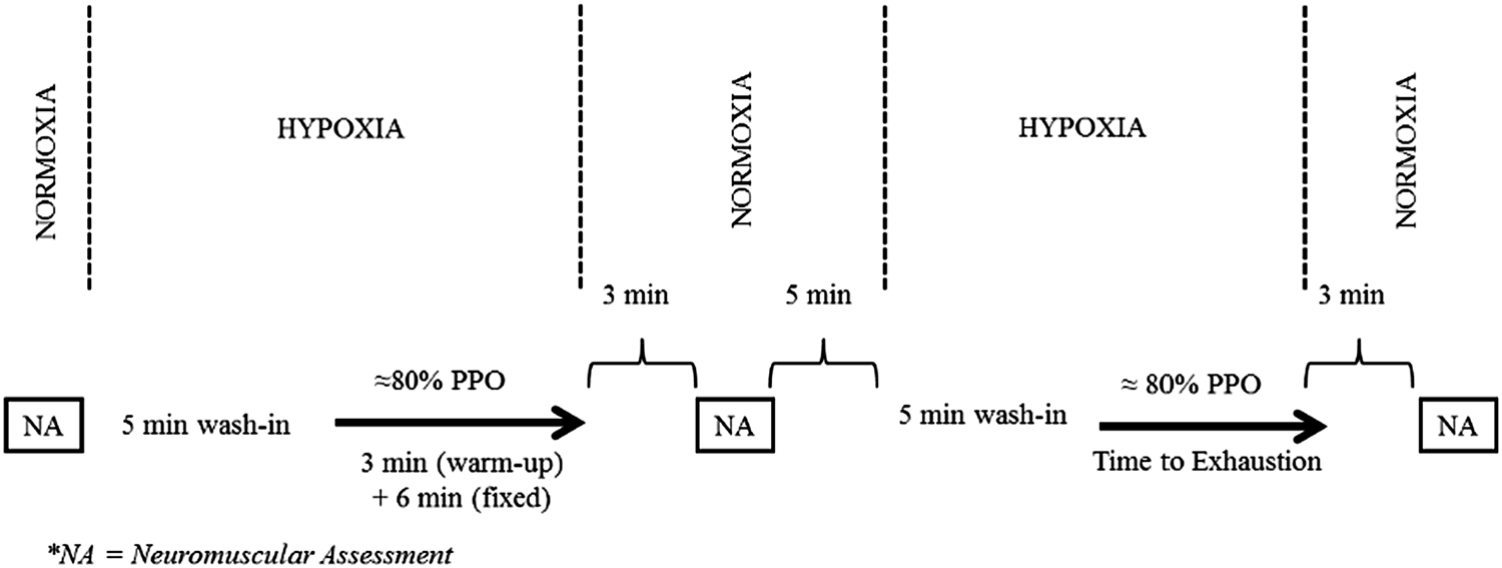
Illustration of the testing protocol during the experimental visits

During the second and third visits, participants were asked to fill a mood questionnaire (see “Psychological questionnaires” for details) and were given an opaque gelatin capsule containing either placebo (dried milk) (DeMorree et al. 2014) or caffeine (4 mg kg^−1^). This caffeine dosage was used as dosages of 3 to 6 mg kg^−1^ are ergogenic, and to allow for better comparison with a previous study in hypoxia (Fulco et al. 1994). Treatment order was randomly allocated according to balanced permutations generated by a web-based computer program (http://www.randomization.com). After 40 min, to reduce instrumentation time after treatment, the electrodes used for the neuromuscular assessment procedures were placed on the participants. One hour after treatment, participants were asked to fill the mood questionnaire for a second time and a questionnaire on motivation related to the upcoming exercise task (see “Psychological questionnaires” for details). Participants were also asked to try to identify which treatment (i.e., caffeine or placebo) they were given, and then immediately started the testing protocol (Fig. 1). The exercise task consisted of: neuromuscular assessment at rest, a wash-in period of 5-min breathing hypoxic air, a 3-min warm-up cycling at 40% PPO, a fixed period of 6 min (isotime) of cycling exercise in hypoxia at 78 ± 3% PPO (216 ± 37 W), neuromuscular assessment 3 min after the end of the 6-min isotime period, 5-min rest followed by a wash-in period of 5-min breathing hypoxic air, time to exhaustion test cycling in hypoxia at ≈80% PPO (78 ± 3%; 216 ± 37 W), and neuromuscular assessment 3 min after the end of the time to exhaustion test. In both the 6-min isotime period and the time to exhaustion test, participants were instructed to maintain a pedal frequency of 70–75 rpm throughout the test, with exhaustion operationally defined as in the incremental test with verbal encouragement given by a blinded investigator. Time to exhaustion was measured using a chronometer to the nearest 1.0 s, from the indication of the researcher to the participants to start pedaling until exhaustion.

#### Physiological and perceptual responses

At the end of warm-up, at the end of the 6-min isotime period and after the time to exhaustion test, a 20-µl sample of whole fresh blood was taken by pricking the participants’ right thumb and analyzed for lactate concentration (Super GL2, Dr. Müller Gerätebau, Germany). Lactate accumulation was calculated by subtracting the 6-min isotime period and exhaustion values by the sample value collected at the end of the warm-up. Heart rate (HR) (Polar RS800CX, Polar Electro Oy, Kempele, Finland) and index finger oxygen saturation (SpO_2_) from pulse-oximetry (model 9500, Nonin Onyx, Plymouth, MN, USA) were recorded each minute during exercise. Also, during the final 15 s of each minute of exercise, participants were asked to rate their overall perception of effort (as already described) on a large RPE scale displayed in front of them throughout the cycling tests. The same physiological and perceptual responses were measured during the preliminary incremental exercise test.

#### Psychological questionnaires

The Brunel Mood Scale (BRUMS) (Terry et al. 2003) was used to assess mood. This questionnaire, which is based on the Profile of Mood States, contains 24 items (e.g., angry, uncertain, miserable, tired, nervous, energetic) divided into six respective subscales: anger, confusion, depression, fatigue, tension, and vigor. The items are answered on a five-point Likert scale (0 = not at all, 1 = a little, 2 = moderately, 3 = quite a bit, 4 = extremely), and each subscale, with four relevant items, can achieve a raw score in the range of 0–16. Of particular interest in the present study were the subscales for fatigue and vigor, which were measured before and 1 h after placebo or caffeine ingestion (before starting the exercise).

Motivation related to the upcoming exercise task was measured 1 h after placebo or caffeine ingestion using the success motivation and intrinsic motivation scales (Matthews et al. 2001). Each scale consists of 7 items (e.g., “I want to succeed on the task” and “I am concerned about not doing as well as I can”) scored on a five-point Likert scale (0 = not at all, 1 = a little, 2 = moderately, 3 = quite a bit, 4 = extremely). Therefore, total scores for these motivation scales range between 0 and 28.

#### Electromyographic recordings

Throughout the exercise task, the surface electromyographic signal (sEMG) was recorded from the right vastus lateralis muscle. Before placing the Ag/AgCl electrodes (Swaromed Universal, Nessler Medizintechnik, Innsbruck, Austria), the skin was shaved, cleaned with alcohol swabs and dried. Electrodes were placed following the SENIAM recommendations (Hermens et al. 2000). The reference electrode was placed at the patella of the same leg. The sEMG signal was amplified (MP150, Biopac Systems Inc., Santa Barbara, CA, USA) and a frequency band filter ranging from 20 to 500 Hz was applied. The sEMG was digitized with a sampling frequency of 2000 Hz and processed (AcqKnowledge 4.1.1 Software, Biopac Systems^®^, CA, USA) by calculating the root-mean-square (RMS) every minute (last 10 s) of the 6-min isotime period and at 25, 50, 75 and 100% (windows of 10 s) of time to exhaustion for placebo, and the respective data points for caffeine. In addition, the exhaustion value for sEMG RMS was also calculated. These sEMG RMS values during the 6-min isotime period and the time to exhaustion test were normalized to the value measured during the last 10 s of the preceding 3-min warm-up.

#### Neuromuscular assessment

Unilateral maximal voluntary contraction (MVC) torque of the right-leg knee extensors was assessed in isometric condition at rest, 3 min after the end of the 6-min isotime period, and 3 min after the end of the time to exhaustion test. An isokinetic dynamometer (Cybex NORM^®^, Humac, CA, USA) was adjusted to position the knee joint at 75° of flexion (0° = leg fully extended) and hip joint at an angle of 90°. Initially, participants performed a warm-up that included ≈10 submaximal contractions of the knee extensor muscles, and was performed only before the stimulations at rest. After 2 min of the warm-up contractions, the stimulation protocol was initiated. The stimulation protocol consisted of one 5-s MVC with doublet delivered over the isometric plateau (superimposed doublet) and 3-s after the MVC (potentiated doublet). Strong verbal encouragement was given during the MVCs by a blinded investigator.

Electrical stimuli were applied transcutaneously using a constant-current stimulator (model DS7AH, Digitimer, Welwyn Garden City, UK). The femoral nerve was stimulated using a monopolar cathode ball electrode (0.5 cm diameter) pressed into the femoral triangle. The site of stimulation was marked on the skin so that it could be utilized again for the entire stimulation protocol. The anode was a 50-cm^2^ (10 × 5 cm) rectangular electrode (Compex SA, Ecublens, Switzerland) located in the gluteal fold opposite to the cathode. During participants’ first visit, stimulus intensity was determined by administering twitches of progressively increasing current. The optimal intensity of stimulation was reached when an increase in the stimulation intensity did not induce further increase in the amplitude of the twitch torque and the peak-to-peak amplitude of the vastus lateralis M-waves. This intensity was further increased by 25% to ensure that it was supramaximal and then maintained for paired stimulations during the second and third visits. The pulse width was 1 ms and the interval between stimuli in the doublet (paired stimulations) was 10 ms. The interpolated twitch technique method was used for determining the voluntary activation level (VAL) as follows: VAL (%) = [1 − (superimposed doublet amplitude/potentiated doublet amplitude)] × 100. A correction was applied to the original equation when the superimposed doublet was elicited slightly before or after the real maximal voluntary torque. In these cases, voluntary activation level was calculated as [1 − (superimposed doublet amplitude × voluntary torque level just before the superimposed doublet/maximal voluntary torque)/potentiated doublet amplitude] × 100 (Strojnik and Komi 1998).

### Statistical analysis

Unless noted otherwise, data are presented as mean ± SD. Paired *t* test was used to compare motivation, time to exhaustion, rest values from the neuromuscular assessment and end values during the time to exhaustion test for RPE, sEMG RMS, HR and SpO_2_ between placebo and caffeine conditions. A two-way repeated measures analysis of variance (ANOVA) with factors time and condition (placebo vs. caffeine) was used for all the other comparisons. The 6-min isotime period and time to exhaustion test were analyzed separately, except for the neuromuscular parameters which involved three data points for the time factor (rest, after the 6-min isotime period, and after exhaustion). For the BRUMS subscales, the time factor data points were before and after pill ingestion. For lactate accumulation, the time factor data points were after the 6-min isotime period and after exhaustion. If a significant time × condition interaction was revealed, post hoc analysis was conducted using paired *t* tests with Holm–Bonferroni stepwise correction method (Holm 1979) to identify the pairwise differences. In these cases, *P* values already adjusted are shown. When time × condition interaction was not significant, a significant main effect of time was followed up using Bonferroni adjustment. Sphericity was tested using Mauchly’s test, and in cases of violation the Greenhouse–Geisser correction was applied. Effect sizes are reported as cohen’s *d* for *t* tests and partial eta squared (*ƞ*_P_^2^) for ANOVA. During the time to exhaustion tests, the following data points for the variables RPE, sEMG RMS, HR and SpO_2_ were compared: (i) the value at the first minute for both conditions (placebo vs. caffeine); (ii) the value closest to 50% of time to exhaustion for placebo and the value at the same absolute time in the caffeine condition; and (iii) the values from the absolute time which occurred for both (placebo and caffeine). Thus, we obtained three values at the same absolute times in both conditions, in addition to the exhaustion values. Due to technical problems, one participant was excluded from the sEMG RMS and HR analysis. Significance was set at *P* < 0.05 (two-tailed) for all analyses, which were conducted using the Statistical Package for the Social Sciences 17.0.0 (SPSS Inc., Chicago, IL, USA).

## Results

### Incremental exercise test in hypoxia

During the incremental exercise test in hypoxia, participants achieved, on average, a PPO of 275 ± 38 W and a VO_2max_ of 51 ​± 5 ml kg^−1^ min^−1^. End values of RPE, lactate concentration, HR and SpO_2_ were 18 ± 1, 13 ± 2 mmol l^−1^, 179 ± 10 bpm, and 81 ± 5%, respectively.

### Effects of caffeine on motivation and mood

**Fig. 2:**
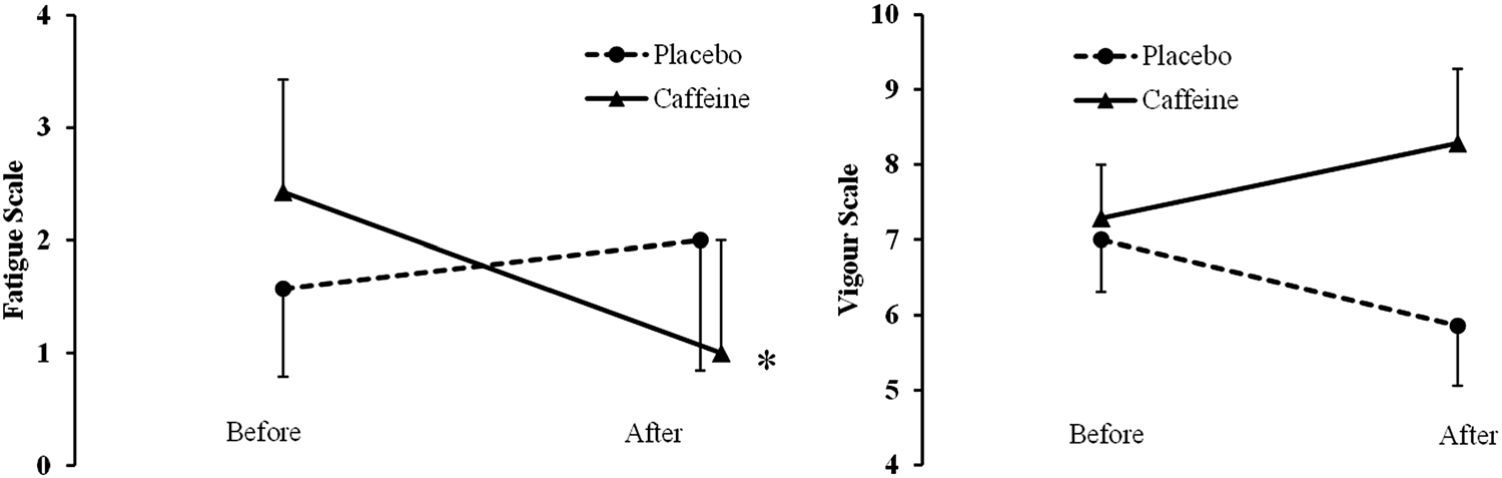
Effects of caffeine on the BRUMS subscales of fatigue and vigor. Values are before and 1 h after pill ingestion (caffeine vs. placebo). *Significant reduction (*P* < 0.05). Data are presented as mean ± SEM

Only 2 of the 7 participants were able to correctly identify the treatment (caffeine vs. placebo). Success motivation (caffeine 17.7 ± 6.2, placebo 17.3 ± 5.8) and intrinsic motivation (caffeine 9.1 ± 0.9, placebo 9.7 ± 1.9) related to the upcoming exercise task did not differ significantly between conditions. The BRUMS questionnaire revealed a significant time × condition interaction (*F*_(1,6)_ = 6.34, P = 0.045, *ƞ*_P_^2^ = 0.51) in the subscale fatigue. Post hoc comparisons revealed a significant decrease in subjective fatigue 1 h after caffeine ingestion (*P* = 0.031) (Fig. 2). The subscale vigor did not present any significant main effect or interaction (Fig. 2).

### Effect of caffeine on performance during high-intensity cycling exercise in hypoxia

**Fig. 3:**
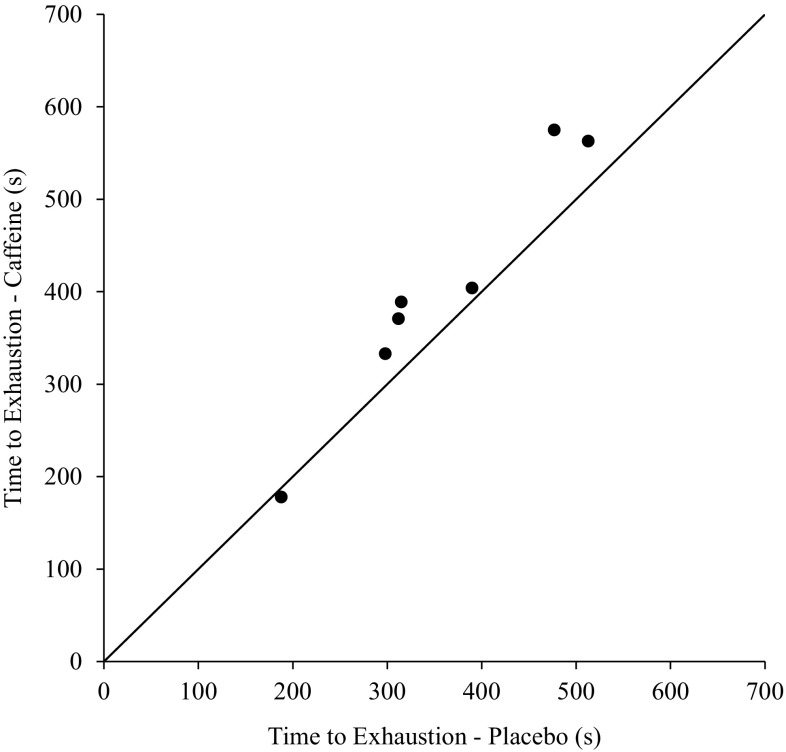
Scatterplot of individual times to exhaustion in the caffeine condition and in the placebo condition. The points above the identity line represent an improved high-intensity cycling exercise performance in the caffeine condition compared to placebo

Time to exhaustion (Fig. 3) was significantly longer in the caffeine condition (402 ± 137 s) compared to placebo (356 ± 112 s) (*t*_(6)_ = −3.32, *P* = 0.016, *d* = 1.26). Individual times to exhaustion were longer in the caffeine condition in six of the seven participants (Fig. 3).

### Effects of caffeine on perceptual and physiological responses during high-intensity cycling exercise in hypoxia

**Fig. 4:**
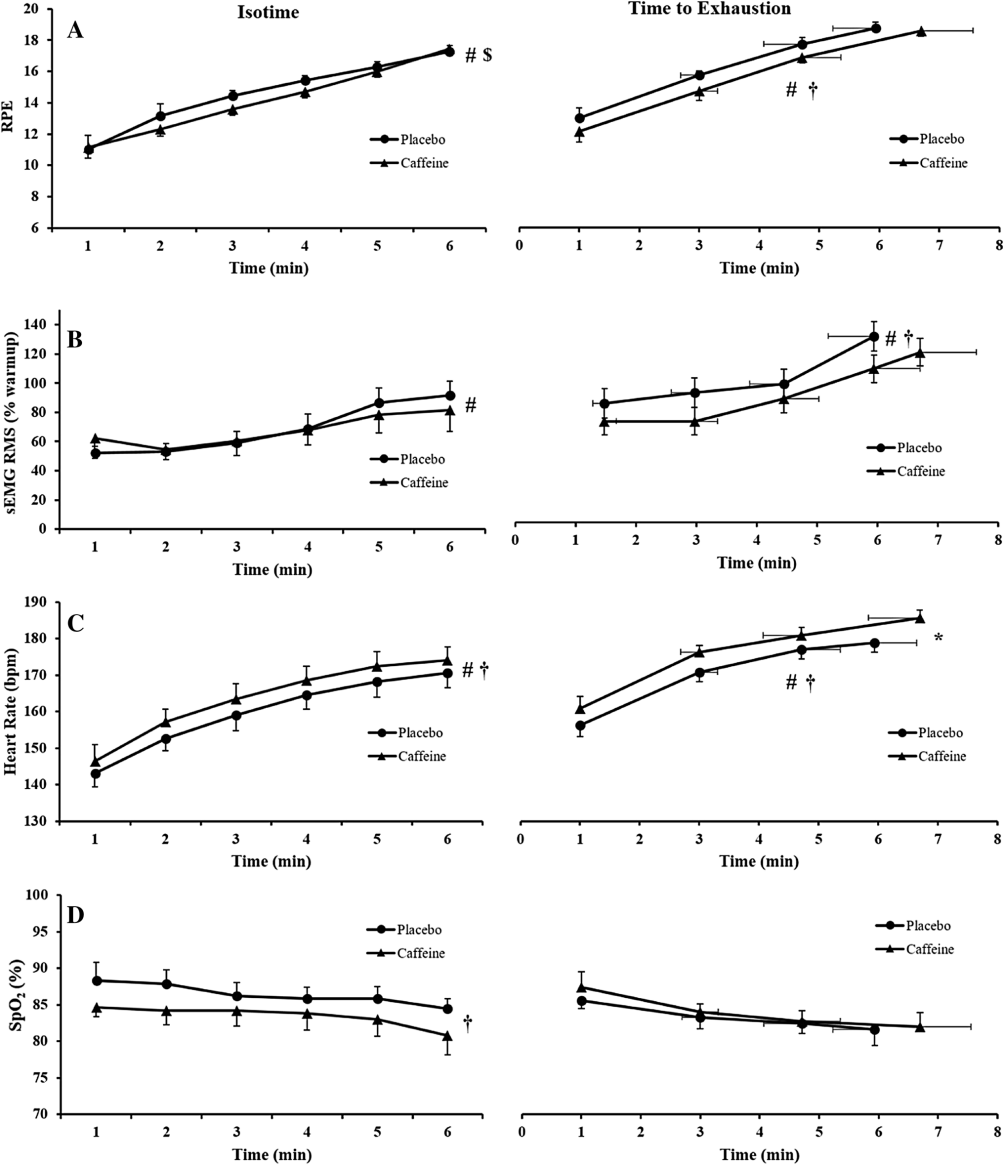
Effects of caffeine on perceptual and physiological responses during high-intensity cycling exercise (6-min isotime period and time to exhaustion test) in hypoxia. **a** RPE. **b** sEMG RMS. **c** Heart rate. **d** SpO_2_. *Significant difference between caffeine and placebo (*P* < 0.05). ^#^Significant main effect of time (*P* < 0.05). ^†^Significant main effect of condition (*P* < 0.05). ^$^Significant interaction (*P* < 0.05). Data are presented as mean ± SEM

During both the 6-min isotime period and the time to exhaustion test, RPE, sEMG RMS and HR increased significantly over time (all main effects of time, *P* < 0.012) (Fig. 4). For the 6-min isotime period, we found a significant time × condition interaction for RPE (*F*_(5,30)_ = 2.77, *P* = 0.036, *ƞ*_P_^2^ = 0.32). However, post hoc analysis did not detect any differences (lowest *P* value was 0.27 for minute 3). During the time to exhaustion test, however, RPE was significantly lower in the caffeine condition compared to placebo (main effect of condition, *F*_(1,6)_ = 6.81, *P* = 0.04, *ƞ*_P_^2^ = 0.53) (Fig. 4a). During the time to exhaustion, sEMG RMS was significantly lower in the caffeine condition compared to placebo (main effect of condition, *F*_(1,5)_ = 6.77, *P* = 0.048, *ƞ*_P_^2^ = 0.58) (Fig. 4b). For HR values, we found a significant main effect of condition for both the 6-min isotime period (*F*_(1,5)_ = 10.12, *P* = 0.024, *ƞ*_P_^2^ = 0.67) and the time to exhaustion test (*F*_(1,5)_ = 9.79, *P* = 0.026, *ƞ*_P_^2^ = 0.66) (Fig. 4c) with significantly higher values in the caffeine condition compared to placebo. While during the 6-min isotime period SpO_2_ was significantly lower in the caffeine compared to placebo (main effect of condition *F*_(1,5)_ = 11.57, *P* = 0.019, *ƞ*_P_^2^ = 0.70), we found no differences during the time to exhaustion test (Fig. 4d). End values for the time to exhaustion test were only significantly different for HR, which presented higher values for caffeine (185 ± 6 bpm) than placebo (179 ± 7 bpm) (*t*_(5)_ = −4.58, *P* = 0.006, *d* = 1.87). Lactate accumulation increased over time (main effect of time, *F*_(1,6)_ = 32.73, *P* = 0.001, *ƞ*_P_^2^ = 0.85) with no significant main effect of condition. Values for placebo and caffeine conditions were 7.5 ± 3.3 vs. 8.8 ± 2.3 mmol l^−1^ after the 6-min isotime period and 10.5 ± 1.6 vs. 12.74 ± 2.4 mmol l^−1^ after the time to exhaustion test, respectively.

### Effects of caffeine on neuromuscular fatigue induced by high-intensity cycling exercise in hypoxia

**Fig. 5:**
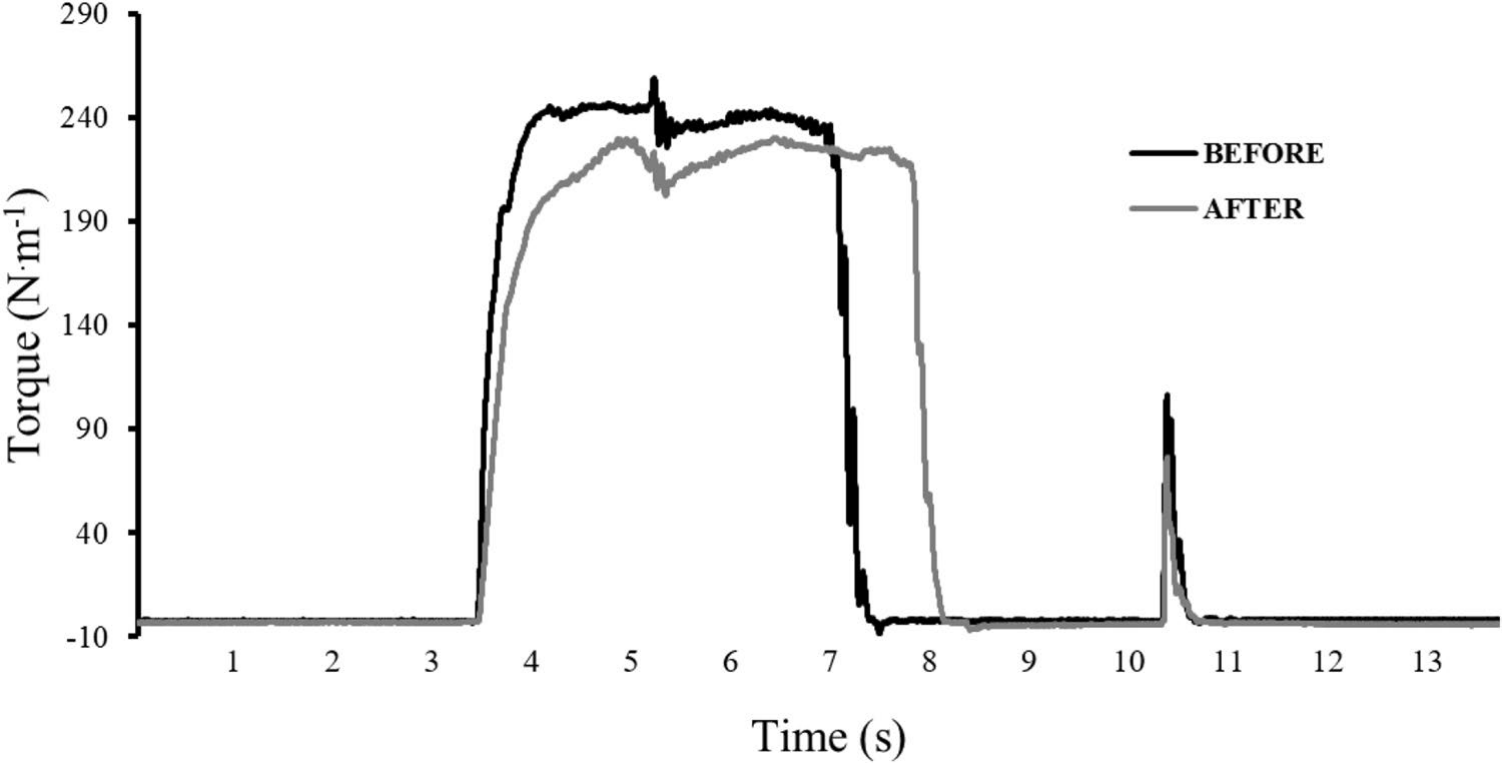
Original torque recordings from one participant related to the measurements of central (superimposed doublet over the maximal voluntary contraction) and peripheral (potentiated doublet at rest) fatigue, both before the exercise task and after exhaustion

**Fig. 6:**
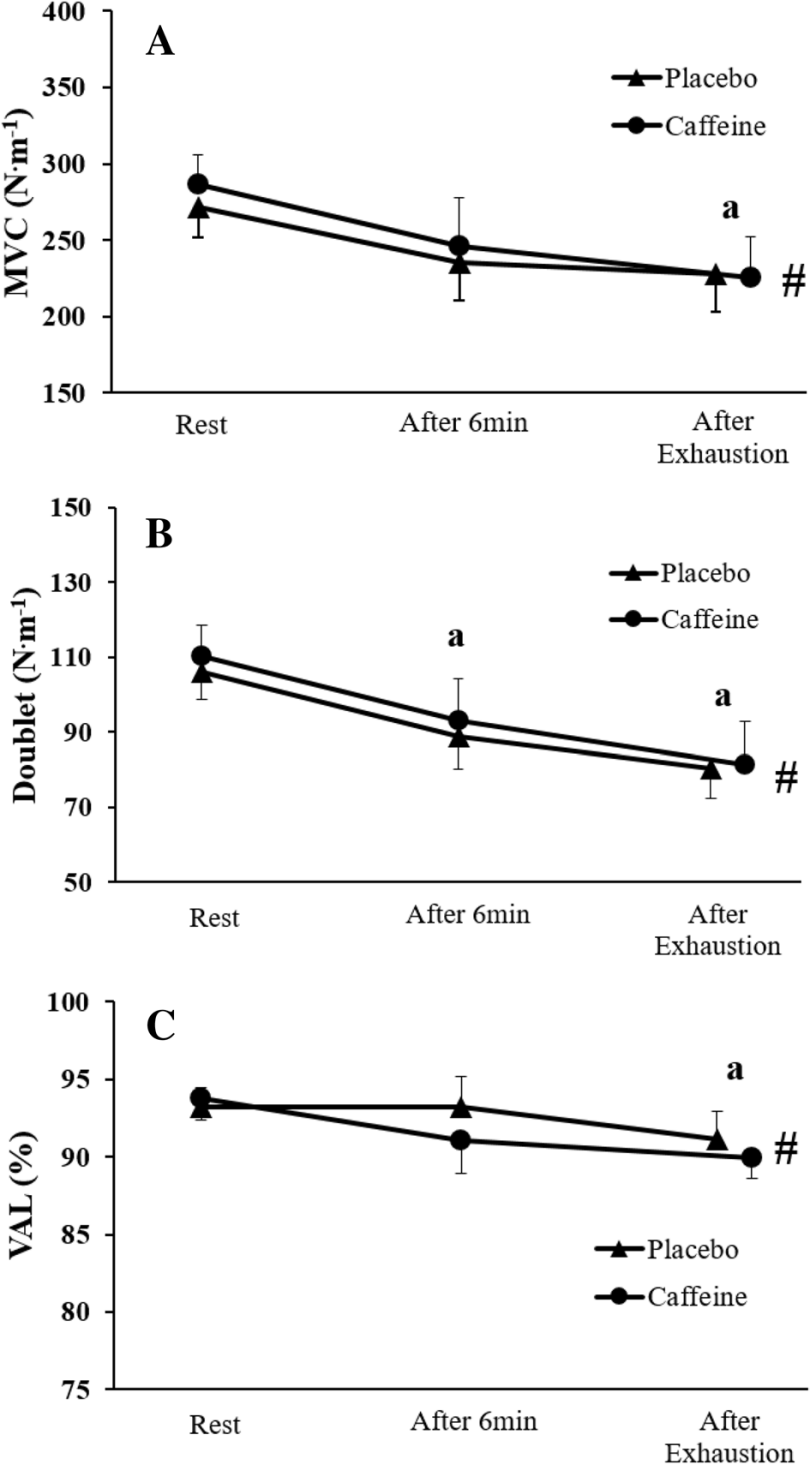
Effects of caffeine on neuromuscular fatigue induced by high-intensity cycling exercise (after the 6-min isotime period and after the time to exhaustion test) in hypoxia. **a** MVC. **b** Peripheral fatigue (doublet). **c** Central fatigue (VAL). ^#^Significant main effect of time (*P* < 0.05). ªSignificant difference from the rest value (*P* < 0.05). Data are presented as mean ± SEM

A typical trace from one participant is illustrated in Fig. 5. MVC torque (*F*_(2,12)_ = 8.08, *P* = 0.006, *ƞ*_P_^2^ = 0.57), doublet (*F*_(2,12)_ = 21.97, *P* < 0.001, *ƞ*_P_^2^ = 0.79) and VAL (*F*_(2,12)_ = 5.15, *P* = 0.024, *ƞ*_P_^2^ = 0.46) presented a significant main effect of time (Fig. 6). MVC torque decreased by 13.4 ± 17.5 and 14.1 ± 19.7% from rest to isotime, and by 16.0 ± 16.6 and 21.4 ± 17.7% from rest to exhaustion in the placebo and caffeine conditions, respectively. Follow-up tests revealed significant reductions in MVC torque only from rest to exhaustion (*P* < 0.05). Doublet decreased by 16.2 ± 11.0 and 15.9 ± 16.6% from rest to isotime, and by 24.3 ± 10.5 and 26.4 ± 22.0% from rest to exhaustion in the placebo and caffeine conditions, respectively. Follow-up tests revealed significant reductions in doublet from rest to isotime (*P* < 0.05) and from rest to exhaustion (*P* < 0.01). Lastly, VAL decreased by 0.0 ± 3.0 and 2.7 ± 4.4% from rest to isotime, and by 3.0 ± 2.3 and 3.8 ± 5.1% from rest to exhaustion in the placebo and caffeine conditions, respectively. Follow-up tests revealed significant reductions in VAL only from rest to exhaustion (*P* < 0.01). Absolute values are presented in Fig. 6.

## Discussion

The main finding of this study is that the effect of caffeine on performance during high-intensity cycling exercise in moderate hypoxia does not seem to be associated with a significant reduction in neuromuscular fatigue. In fact, contrary to our hypotheses, the MVC loss, central fatigue and peripheral fatigue induced by high-intensity cycling exercise in moderate hypoxia measured 3 min after exhaustion were not reduced by caffeine ingestion. Our study was the second to measure the effects of caffeine on these neuromuscular parameters before and after dynamic exercises in hypoxia and, therefore, comparisons are difficult. Eaton et al. (2016), the only other study with a similar testing protocol, did not find any difference in neuromuscular parameters (MVC and twitch torque, and central activation ratio) when comparing the caffeine only and placebo conditions after an exercise task designed to mimic the running profile of team sport athletes. This study, which involved other conditions such as caffeine plus essential amino acids, presented a few moderating factors such as heat (30 °C) and prior ingestion of carbohydrates in addition to hypoxia (Eaton et al. 2016). Utilizing a similar testing protocol in normoxia, Black et al. (2015) investigated the effects of caffeine (5 mg kg^−1^) on MVC loss, central fatigue and peripheral fatigue after both arm and leg cycling exercise with a period of 30 min at a constant intensity (60% *V*O_2_max), followed by a 10-min time trial. Similar to our results, they found that caffeine reduced perception of effort and pain, and improved performance despite no effect on neuromuscular fatigue when compared to placebo. It is noteworthy, however, that neuromuscular assessments were made only 20 min after time-trial completion (Black et al. 2015). Interestingly, in their additional study, Black et al. (2015) found no differences in perception of effort, pain and time to exhaustion at exercise intensities closer to the ones used in the present study after caffeine ingestion. This is unexpected, since the literature has shown a rather consistent effect of caffeine on perception of effort (Doherty et al. 2004; Doherty and Smith 2005) and on endurance performance (Doherty and Smith 2004; Astorino and Roberson 2010). The only other study investigating the effects of caffeine on neuromuscular fatigue during dynamic exercise reported some contrasting results (Cureton et al. 2007). Caffeine improved performance during the exercise task (cycling 120 min between 60 and 75% *V*O_2_max + 15-min time trial) and attenuated the MVC loss measured 20 min after exercise termination, as well as avoided the development of peripheral fatigue, assessed 7.5 min after exercise termination. However, as also acknowledged by the authors (Cureton et al. 2007), it is difficult to isolate the ergogenic effects of caffeine on neuromuscular fatigue, as the caffeine condition also included ingestion of CHO, taurine, carnitine, vitamins and sucralose. In addition, it is difficult to distinguish between the effects of these substances on fatigue development or on the recovery process, given the long recovery time between exercise termination and neuromuscular assessment.

Our sEMG RMS data from the vastus lateralis revealed a significantly lower sEMG amplitude in the caffeine condition during the time to exhaustion test, but not during the 6-min isotime period, in the caffeine condition when compared to placebo despite being performed at the same absolute power output and pedaling cadence (Fig. 4b). However, as central and peripheral fatigue did not differ between conditions, these results are difficult to explain. When neuromuscular fatigue (central and peripheral fatigue) is unaltered, no difference in sEMG amplitude during exercise is expected. Although we have not normalized sEMG RMS data by the maximal M-wave (see “Limitations”), two studies involving mental fatigue confirmed this expectation (Pageaux et al. 2013; Rozand et al. 2014), while another study found a dissociation between sEMG amplitude and neuromuscular fatigue (Pageaux et al. 2015), similar to our results. A speculative hypothesis may be an alteration in motor control between conditions (placebo vs. caffeine) after certain degree of fatigue occurred during the time to exhaustion test (Budini et al. 2014). However, a measure of muscle co-activation (e.g., biceps femoris) would be necessary to strengthen this hypothesis. In addition, the lack of normalization of the sEMG RMS data by the M-waves, as well as measurement of sEMG and neuromuscular fatigue in different conditions (exercise in hypoxia vs. after exercise in normoxia, respectively), further complicates this interpretation.

Despite no positive effects on neuromuscular fatigue, caffeine led to a significant 12% improvement in time to exhaustion during high-intensity cycling exercise in conditions of moderate hypoxia equivalent to 2500 m altitude. This finding corroborates previous studies on the ergogenic effect of caffeine on endurance performance in hypoxia. Stadheim et al. (2015) found that caffeine increases time to exhaustion during a high-intensity double poling exercise by 20% at a simulated altitude of 2000 m above sea level. With a high-intensity cycling test similar to ours, Fulco et al. (1994) found a significant 54% improvement in time to exhaustion after caffeine ingestion in conditions of acute hypoxia at an altitude of 4300 m above sea level. With regard to time-trial performance, Berglund and Hemmingsson (1982) studied cross-country skiers at 2900 m and found that caffeine induces a significant 1.7% reduction in performance time during a 21-km time trial. In a more recent investigation of cross-country skiers, Stadheim et al. (2015) found that caffeine induces a non-significant 0.9% reduction in performance time during a double poling 8-km time trial at a simulated altitude of 2000 m above sea level.

The most likely explanation for enhanced endurance performance in hypoxic conditions is the reduction in perception of effort induced by caffeine (Fig. 4a). Such perceptual effect of caffeine has been previously observed in both normoxia (Doherty et al. 2004; Doherty and Smith 2005) and hypoxia (Berglund and Hemmingsson 1982; Fulco et al. 1994; Stadheim et al. 2015), and it is considered one of the main mechanisms through which caffeine improves endurance performance in normoxia (Doherty and Smith 2005). These findings fit with the psychobiological model of endurance performance (Smirmaul et al. 2013). This model postulates that exhaustion is not directly caused by neuromuscular fatigue but rather by a decision-making process based on perception of effort and potential motivation (defined as the maximum effort one is willing to exert in order to succeed in the task) (Marcora et al. 2008; Marcora and Staiano 2010; Smirmaul et al. 2013). Therefore, any intervention that reduces perception of effort and/or increase potential motivation should increase time to exhaustion even in the absence of a significant reduction in neuromuscular fatigue. Similar scores for RPE at exhaustion and the motivation scales suggest that caffeine did not increase potential motivation. However, the likely lower RPE during the 6-min isotime period (significant time × condition interaction, but no significant differences detected by the post hoc analysis, see “Results” section) and during the time to exhaustion test (significant main effect of condition) shows that perception of effort was likely reduced by caffeine ingestion, with consequent delay of the time at which most participants reached their perceived maximal effort and decided to stop exercising (Fig. 4a). The present study design, however, employed a 5-min rest followed by a wash-in period of 5-min breathing hypoxic air after the neuromuscular assessments (between the 6-min isotime period and the time to exhaustion test), and it is difficult to assure that the starting states of the neuromuscular parameters for the time to exhaustion test were similar between conditions. If fatigue had better recovered in the caffeine condition during this recovery period, the lower RPE may have reflected the lower extent of neuromuscular fatigue. In addition, as our peripheral fatigue results did not demonstrate differences between conditions despite increased time to exhaustion after caffeine ingestion, other theoretical models such as the “critical peripheral fatigue threshold model” (Amann et al. 2013) cannot be discarded as an alternative explanation for the ergogenic effect of caffeine. However, there is evidence that a critical peripheral fatigue threshold does not limit endurance performance (Smirmaul and Dantas 2011; Johnson et al. 2015).

Although measured at different time points and environmental conditions (after exercise in hypoxia vs. at rest in normoxia), neuromuscular fatigue occurred to a similar extent in the caffeine and placebo conditions. Thus, the observed reduction in perception of effort after caffeine ingestion is not likely to be explained by a reduction in the central motor command required to compensate for neuromuscular fatigue (DeMorree et al. 2012). Instead, the caffeine-induced reduction in perception of effort may result from a direct effect of caffeine on the brain that can reduce the activity of premotor and/or motor areas of the cortex even when force output stays constant (DeMorree et al. 2014). A direct effect of caffeine on the brain of our participants is indirectly corroborated by the significant effect of caffeine on subjective fatigue measured at rest via a mood questionnaire (Fig. 2). In addition, caffeine increased HR, an effect commonly observed during exercise in normoxia and corroborated in hypoxia (Stadheim et al. 2015). While SpO_2_ did not differ between conditions during the time to exhaustion test, an unexpected finding was its reduction during the 6-min isotime period after caffeine ingestion. This results is contrary to previous findings showing a caffeine-induced  increase in SpO_2_ during similar submaximal exercise intensities (Chapman and Mickleborough 2009). However, how these caffeine-induced effects on HR, SpO_2_ and ventilation (not measured in the present study) interact to positively or negatively influence respiratory effort and endurance performance at altitude is currently unknown (please refer to Chapman and Mickleborough 2009). Lastly, while the literature has shown mixed results on the effects of caffeine on lactate concentrations (Davis and Green 2009), we found no effect of caffeine ingestion on lactate accumulation.

### Limitations

A few limitations need to be taken into account in evaluating the present study. First, voluntary activation was assessed during an isometric MVC of the knee extensor muscles, which is a different task from cycling. This may have precluded detection of all the specific fatigue processes induced by high-intensity cycling exercise in moderate hypoxia. Second, neuromuscular assessment was performed 3 min after exhaustion, which may have led to an underestimation of the extent of neuromuscular fatigue-induced high-intensity cycling exercise in moderate hypoxia (Froyd et al. 2013). However, using a similar recovery period, previous studies were still able to find differences in the extent of neuromuscular fatigue (Amann et al. 2006; Goodall et al. 2012). Furthermore, a study involving cycling exercise performed in similar conditions of moderate hypoxia (FIO_2_ = 0.15) has shown that neuromuscular fatigue does not recover within 1–4 min after exercise termination (Dahlstrom et al. 2013). Third, we did not measure M-waves during the experimental visits, not allowing for normalization of the sEMG RMS data to the M-waves and limiting our control over possible changes in neuromuscular junction and sarcolemma excitation. Fourth, although all procedures were performed in a similar way for both conditions (placebo × caffeine), we assessed neuromuscular fatigue parameters in normoxia. This study design has been used previously in studies of neuromuscular fatigue during high-intensity cycling exercise in hypoxia (Amann et al. 2006; Romer et al. 2007). However, it is not possible to rule out a potential recovery from the specific effects of hypoxia. Lastly, the lack of respiratory variables limits our understanding of the SpO_2_ results, and future investigations may further investigate this issue.

## Conclusion

The positive effect of caffeine on high-intensity cycling exercise performance in moderate hypoxia seems to be mediated by the caffeine-induced reduction in perception of effort and improved mood, rather than reduced neuromuscular fatigue. These findings provide preliminary information to physiologists regarding the mechanisms underlying the ergogenic effect of caffeine during exercise in hypoxic conditions. They also provide further evidence to coaches, athletes, mountaineers and soldiers operating at high altitude that caffeine supplementation may reduce, in part, the negative effects of hypoxia on perception of effort and endurance performance.

## Abbreviation

ANOVA: Analysis of variance
BRUMS: Brunel mood scale
FIO_2_: Fraction inspired oxygen
HR: Heart rate
MVC: Maximal voluntary contraction
*ƞ*_P_^2^: Partial eta squared
PPO: Peak power output
RMS: Root-mean-square
RPE: Rating of perceived exertion
sEMG: Surface electromyographic signals
SpO_2_: Oxygen saturation
VAL: Voluntary activation level
*V*O_2_max: Maximal oxygen consumption
